# Physician perspectives on the DNR order in Turkey: A survey of physicians in Internal Medicine

**DOI:** 10.4314/ahs.v23i1.71

**Published:** 2023-03

**Authors:** Güvercin Cemal Hüseyin, Kurtoğlu Ayşe Pelin, Yazici Güvercin Ayşe Canan

**Affiliations:** 1 Dokuz Eylul University Faculty of Medicine, History of Medicine and Ethics; 2 Balikesir Provincial Health Directorate; 3 Izmir Tinaztepe University Faculty of Medicine, Biostatistics

**Keywords:** Do not resuscitate (DNR), physicians' views, legal regulation

## Abstract

**Background:**

Do not resuscitate (DNR) is a controversial ethico-legal issue and there is no legal regulation in Turkey. Evaluating the physicians' views on DNR is critical to the current problems and contributes to legal regulation.

**Objectives:**

To examine the views of intensive care unit residents on DNR and the sociocultural and occupational factors affecting them.

**Methods:**

The research is a descriptive cross-sectional study. The sample of the study consists of 203 residents of internal medicine working in the intensive care unit in a university hospital. A questionnaire form was used as a data collection tool.

**Results:**

62.6% of the physicians know that there is no legal regulation regarding DNR in Turkey, and 14.3% think that DNR is performed. Female physicians approve of DNR at a higher rate than men (p<0.01). Physicians with more experience in the profession stated that not all patients should be performed cardiopulmonary resuscitation (p<0.01), and DNR should be a right (p<0.05). The vast majority of physicians stated that DNR should be legal (88.1%) and should be included in residency training (85.6%).

**Conclusions:**

It is necessary to establish legal regulations on DNR and implement residency training programs that will ensure the continuous professional development of physicians.

## Introduction

In this century, along with rapidly accelerating scientific breakthroughs, human life is prolonging with advances in the medicine. At the same time, the prevalence of cancer and degenerative diseases increases with age. These diseases, which require long-term care and treatment, lead to an increase in the patients who receive treatment in intensive care conditions and spend the last period of their life in intensive care units, especially in developed countries. For this reason, intensive care units have an important role among the hospital units where the decision on how to end life is made.^1^

The patient's lives given life support in intensive care units generally end in two ways; withdrawing or withholding life-sustaining treatment. Withdraw treatment; the patient's inability to benefit from a previously started treatment or intervention or stopping supply at one's request. On the other hand, withholding treatment considers the clinical condition of the patient and the quality of life afterward, and then no further treatment or aggressive intervention is initiated. As a result-of-life the decisions of both withholding and withdrawing are similar, and opinions are stating that there is no ethical difference between them. Nevertheless, healthcare professionals prefer to withhold rather than withdraw.^2^,^3^.

The concept of “Do not resuscitate (DNR)” is under the umbrella of withholding treatment.^3^ DNR has been defined as the decision not to perform cardiopulmonary resuscitation (CPR) in the respiratory or cardiac arrest if the patient's survival causes suffering and futility because of not benefiting from the treatment or not stopping the disease progression. 4

For the patient, end-of-life decisions are closely related to the DNR and in general right to refuse treatment, the autonomy of will, and the right to self-determination. Despite the importance of patient autonomy, the patient's request for end-of-life wishes has not been abided by the paternalistic approach.^5^–^7^ However, the rate of CPR performed in intensive care units from the 1980s to the 1990s decreased, the termination of treatment increased.^8^ In many European countries and the US, passive euthanasia limiting or withdrawing life support is legal, and the prevalence of these practices is increasing. There is a remarkable cultural difference between the East and the West in terms of attitudes and practices.^9^–^14^

The DNR subject is discussed over different approaches and assumptions because of the not included as a clear legal regulation in Turkish Law. According to the Convention for the Protection of Human Rights and Dignity of the Human Being with regard to the Application of Biology and Medicine (also known as the Oviedo Convention) which has been our national law since 2003; for any medical intervention, the informed consent of the individual should be obtained, and if the person is unable to give consent, the advanced directive should be considered. As stated in this legal regulation, CPR should not be performed if the person requests DNR and rejects the resuscitation.^15^ Accordingly, the Patient Rights Regulation, the patient has the right to refuse or stop the treatment at his/her own risk for the negative consequences.^16^ However, this regulation also states that nothing can be done or demanded that may cause death or is life-threatening except for diagnosis, treatment, or protection. In addition, it has been clearly emphasized that the prohibition of euthanasia and the right to life cannot be given up. Therefore, a contradictory situation has arisen. If refusing treatment will lead to death, the patient's request will not be valid, and the right to refuse treatment is bound to the condition of not causing death. This regulation does not allow DNR to perform. Although there is a conflict between the Oviedo Convention and the Patient Rights Regulation, the Oviedo Convention is valid in terms of the principle of the law of international regulation over national law in Article 90 of the Constitution. However, a decision of the Supreme Court emphasized that such practices have been punished as a violation of the prohibition of euthanasia, stated that even with the individual's consent, he did not have unlimited power of disposition on the rights related to bodily integrity.^17^ Despite the controversial and risky legal situation, studies carried out in Turkey show that a percentage as significant as 40.0-65.9% of physicians in this area have practiced limiting or withdrawing treatment. Previous studies have focused only on religious views rather than social and cultural factors that may affect the decision-making process of physicians.^18^–^20^

Despite the DNR subject is widely accepted and approached moderately by physicians, it is also a controversial issue in ethics and law. Putting forth the current approach of young physicians who serve in intensive care units on this issue will contribute to the discussions in this area and may also be a guide for legal regulations.

The aim of this study is to evaluate the relationship between the opinions and thoughts on DNR of resident physicians working in the intensive care unit and some socio-cultural parameters.

## Material and Method

The study was planned as a descriptive cross-sectional study. The sample study consists of all the resident physicians (briefly mentioned as a physician in the text) who graduate medical school and take the examination for specialty in medicine and training of internal medicine sciences. They have worked at the intensive care units of Dokuz Eylül University Medical Faculty Hospital as doctors who may have experience with a DNR patient. After data control, 203 forms were included in the study. As a data collection tool, a questionnaire form requesting the demographic information of physicians and their thoughts and opinions on DNR was created. The qualitative questionnaire form, 22 questions in which demographic information and information about the subject are assessed, and 46 questions for understanding the thoughts and opinions of physicians, consists of two parts. While inclusion criteria for the study were being resident physician in internal medicine sciences and willingness to participate in the study, exclusion criteria were not agreeing to enter the study.

The study was initiated after obtaining permission from head of the intensive care department, chief physician of the hospital, and than the research ethics committee. Approval was obtained from the Dokuz Eylül University Non-Interventional Research Ethics Committee in October 2018 with the protocol number 3899.

We informed the intensive care physicians about the study, those who agreed to participate in the study filled out the questionnaire after reading and accepting the informed consent form at the beginning of the questionnaire. A face-to-face questionnaire form was distributed in person and participants filled it out themselves. The participants had adequate time to review the consent form, ask questions about the study, and filled the questionnaire form.

### Statistical Analysis

In order to determine relationships between categorical variables Chi-square tests, Likelihood ratio test because of the small expected frequencies in some cells of some contingency tables and Fisher's Exact test were applied. Compliance with the normal distribution of continuous variables that age, years of practice, and years in residency were checked with Shapiro-Wilk test. Homogeneity of groups' variances was checked by Levene's test. If parametric test assumptions are available, two independent group means were compared by Student's t test. If parametric test assumptions are not available, Mann Whitney U test was used for comparisons of two groups medians. The validity of the data set was evaluated by Factor Analysis. The Reliability was determined by Cronbach's Alpha statistics.

Data analyses were performed using the Statistical Package for the Social Sciences, version 19.0^21^. A p value of ≤0.05 was considered statistically significant.

### Findings

The questionnaire was answered by 203 resident physicians at Dokuz Eylül University Hospital in the departments of internal medicine, pulmonary medicine, anaesthesiology and reanimation, emergency medicine, neurology, and cardiology and also work in the intensive care units. The explanation rate of the total variance in the study was 66.832% and the Cronbach alpha value for the total was obtained as 0.713.

The socio-demographic characteristics of the physicians in the study are included in Table 1.

**Table 1:** Socio-demographic characteristics

Variable	Category	n (%)
Gender	Female/ Male	100 (49.3) / 103 (50.7)
Marital status	Married/ Single	77 (37.9) / 126 (62.1)
Department	Internal medicine / Other*	110 (54.2) / 93 (45.8)
Years of practice	5 years or less / More than 5 years	123 (60.9) / 79 (39.1)
Years in residency	2 years or less / More than 2 years	104 (52.0) / 96 (48.0)

* Emergency medicine (n = 31, 15.3%), Anaesthesiology and reanimation (n = 30, 14.8%), Neurology (n = 8, 3.9%), Pulmonary medicine (n = 8, 3.9%), Cardiology (n = 10, 4.9%), Physician from another department (n = 6, 3.0%)

The mean age and standard deviation of the respondents were 29.09 ± 3.41; the median age is 28 (min 24; max 46). 49.3% of the physicians are women and 50.7% are men. Mean age± standard deviation and median value for males were 29.13 ± 3.63 and 28.0 (min 24; max 46), and for females were 29.05 ± 3.21 and 28.0 (min 24; max 39). The mean, standard deviation and median of the time they worked as a physician were for men 6.0 ± 3.94; 5.00 (min 1 max 24), and for women 5.54 ± 3.15; 5.00 (min 1, max 15) respectively. The longest settlement of almost all of the participants (94.6%) is urban settlements. The top three geographic regions with the longest settlement duration; Aegean (47.3%), Marmara (14.8%) and Central Anatolia (14.3%). About 2 out of the 3 physicians live in western part of Turkey, which is considered to be more secular.

In this study, although most of the physicians stated that they were caring for terminal patients and performed CPR, and half of them cared patients who requested DNR, the rate of physicians who knew that there was no legal regulation on this issue was 62.6%. It is a striking finding that one out of every three physicians are unaware of the legal situation in a critical issue such as DNR. While almost all of the physicians took ethics/medical deontology lessons in undergraduate education, approximately one out of every four physicians stated that they read a literature on DNR, and only one of six physicians attended a meeting on the subject. 14.3% of the physicians believe that DNR orders apply in Turkey.

In present study, the rate of physicians having accurate information about the legal regulation in Turkey on DNR was found to be higher among physicians who lived in the western regions, the highest being in the Aegean region (p < 0.01). The average number of years in practice was found to be significantly higher in physicians who correctly answered the question about the legal situation in Turkey (p < 0.05). It was also found that physicians, with more than 2 years of residency period, performing CPR, and caring for terminal patients had higher rates of correct response regarding the legal status of DNR. (p< 0.01; p <0.05; p <0.001; respectively).

It was observed that the rate of monitoring the literature on DNR was higher in physicians practicing in internal medicine residency or had a patient who requested DNR (p < 0.05 and p <0.001, respectively). Interestingly, internal medicine physicians have the most negative view of DNR.

The mean age, number of years in practice and residency are statistically significantly higher for physicians with more experience in caring for terminal patients, and performing CPR. (Respectively p < 0.001; p <0.001; p <0.001 and respectively p <0.001; p <0.001; p <0.001). These groups of physicians think that the DNR request of the patient is not abided by in Turkey. (p <0.001; and p <0.001, respectively). It is possible to say that as the experience of physicians increases, they gain a more realistic approach to DNR.

Approximately 3 out of 4 physicians who had experience in patients' DNR requests, terminal patient care, and performing CPR think that the DNR request of the patient is not abided by in the country. Female physicians approve of DNR more than men (p <0.01). Physicians who have the correct knowledge about the legal regulation on DNR, those with a high number of years in practice, and those with higher parental education think that the DNR request should be a right and physicians should not perform CPR on all patients. (p = 0.001; p < 0.05; p = 0.05; and p = 0.01, p = 0.01; p = 0.05, respectively).

Physicians with higher mean age, number of years in practice, and residency stated that the physician and nurse should be the first to bring up the idea of DNR. (p = 0.05; p = 0.01; p = 0.05).

Concern about violence from relatives of patients due to DNR is statistically significantly higher in female physicians and pulmonology physicians. (For two of them p <0.05).

In our study, approximately 3 out of 4 physicians stated that DNR should be a right, the patient should make the final decision on DNR, physicians should abide by the patient's request, and these patients should continue to get palliative care. These physicians believe that the patient's DNR decision should be approved by an authorized institution and that these patients should be cared for by their own physicians. Additionally, physicians give a significantly negative opinion on the application of CPR to all patients and the application of “slow code” to patients who request DNR.

Approximately 9 out of 10 physicians want legal regulations on DNR and DNR to be in the resident training program. Most physicians have a positive attitude towards recommending DNR to their patients, providing counselling when necessary, and asking for DNR for themselves and their relatives. While making the DNR decision, physicians do not take into account factors such as cost, bed occupancy rate, and the social status of the patient. However, they state that they will consider the prognosis of the disease, the patient's being a donor, and the patient's age. Physicians who have treated terminal patients significantly supported discussing DNR with the patient, recommending it to patients/patients' relatives, and taking the DNR decision by physicians. Additionally, they stated that they were less worried about making the DNR decision. On the other hand, physicians who had not cared for a patient requesting DNR said they were more concerned. (p < 0.05; p = 0.05; p = 0.01; p = 0.05; p = 0.05).).

Similarly, physicians with patients requesting DNR are more supportive of raising the topic by the patient first, while inexperienced physicians express that they are more indecisive. (p = 0.01).

Physicians, who stated that they would recommend DNR to their patients, think that they should be the ones making the DNR decision. (p <0.001). Physicians, who want DNR for themselves, stated that the patient's DNR request should be abided by. (p <0.001). Physicians who stated that the DNR request should be abided by said that they could apply slow code to their patients (p < 0.01), and they would not perform CPR if they saw a DNR tattoo on the patient's chest (p < 0.01). Physicians who found it useless to perform CPR on terminal patients stated that they would consider the age of the patient (p< 0.001), the nature of the disease, prognosis (p < 0.01), and whether the patient was an organ donor (p<0.001) in their DNR decision. Most of the physicians who stated that they did not find the DNR application appropriate due to their religious beliefs also said that CPR should be performed on every patient with arrest (p< 0.01). Physicians who disapprove of the DNR decision in terms of ethics stated that they did not find the DNR decision appropriate due to religious belief (p<0.001), they also said that DNR should not be applied even if the patient/relatives requested it (p<0.001) These physicians also expressed the concern that the patient would not receive adequate care after the DNR decision (p < 0.01).

Physicians who approved of DNR (p<0.001) stated that patients and their relatives should receive counseling from trained personnel (p<0.001). Physicians who suggested that the patient be taken to a separate center after the DNR decision stated that the bed occupancy rate or economic cost should also be considered in the DNR decision (p < 0.01, p <0.001, respectively)

Physicians who believe the ethics committee should approve the DNR order stated that they are afraid of legal problems (p<0.001) and violence from patients/relatives (p<0.001) and that patients and relatives should receive counseling from trained personnel in DNR (p <0.001). These physicians stated that the education and social status of the patient should be taken into account when making the DNR decision (p<0.001).

## Discussion

Physicians' attitudes towards DNR are influenced by sociodemographic factors such as age, gender, marital status, parental education level, belief, and cultural environment. In addition, these attitudes can be shaped depending on professional factors such as receiving undergraduate ethics training, specialty, training on DNR, caring for a terminal patient, having a patient requesting DNR, years in practice.

We found that the attitudes and opinions of physicians on DNR were particularly affected by gender and parental education level, but not by parameters such as marital status, geographical origin. Female physicians approve of the DNR decision more than male physicians. In the study of Periyakoil et al., female physicians reported a higher rate of approval than male physicians on advance directives including the DNR decision.^22^ In the study of the İyilikçi, similarly, it was reported that, female physicians disapproved of full life support.^18^ However, in the study of Chen, no statistically significant difference was found between the genders in terms of their views on DNR.^23^ In our study, it was determined that parental education level, which is an indicator of the influence of the family on the development of individual values, is associated with the end-of-life decisions of the physician. Physicians with at least one parent with higher education have a more liberal and positive perspective on DNR. It has been determined that other sociodemographic parameters other than gender and parental education level do not have a statistically significant effect on physicians' perspective on DNR.

In the study of Periyakoil et al., it was determined that physicians' approaches to advance directives, including the DNR decision, differ according to gender, ethnicity and area of specialty, and the positive attitude towards DNR is highest in emergency department physicians. In our study, even though there was no statistically significant relationship between specialty and attitude towards DNR, a higher number of internal medicine physicians had a negative attitude towards DNR. While in the study of Periyakoil, 88.3% of the physicians stated that they wanted DNR for themselves, in our study, this rate was relatively lower at 68.7%.^22^

In our study, 10.4% of the physicians stated that they did not find the DNR decision correct due to professional, 11.4% ethical, and 8.0% religious' reasons. According to The Religious Affairs Administration in Turkey, from an Islamic perspective, although active euthanasia is prohibited, if there is no medical hope to survive life support unit may be disconnected, but the nutrition supply should continue. In case of brain death, cessation of life support and organ donation are supported.^24^,^25^ A multi-centered study in Europe shows that regional and religious differences were consequential in end-of-life decisions. According to this study, Catholics, Protestants, and those who do not profess religious beliefs decide to withdraw treatment more than Muslim, Jewish, and Orthodox physicians.^26^ A study with Muslim physicians pointed out that the physician's place of birth, workplace, and experiences was more significant than one's religious belief.^27^ Some studies conducted in the Middle East countries has pointed out that religious beliefs and cultural background were significant in the DNR decision. 12–14,28 Whereas, according to the study of Saeed et al., in which physicians from different countries participated, it was determined that the religious views of Muslim physicians did not have a significant effect on end-of-life decisions. But in these decisions, it was emphasized that the country and social environment where physicians live and get an education have more influence than belief.^27^ We determined in this study that religious beliefs were the least influential among the socio-cultural factors that could affect physicians' decisions on DNR. This result can be accepted as a positive attitude in terms of professional autonomy and clinical independence of physicians. It is ethically significant for most of the physicians to make decisions without being influenced by a belief on an issue related to the phenomenon of death, which is one of the most fundamental issues in religions. The fact that most of the physicians (95.6%) received ethics/ medical deontology lessons in their undergraduate education, which is one of the findings in our study, may have contributed to these attitudes of the physicians. Getting training in end-of-life decisions may change the physicians' perspective about DNR. After the training, changes were observed in the content, time and rates of talking with their patients about end-of-life decisions.^29^,^30^ However, in our study, during the residency training process, the rate of attending a professional meeting about DNR is low. Physicians are trying to close this education gap by themselves and when they have a patient who requests DNR, it is seen that the rate of monitoring the literature on DNR increases significantly. Pointing out both the existence of the problem and the need for training, 85.6% of the physicians want DNR as a subject is in the specialty training program.

The parameters that constitute the professional experience of physicians such as age, experience in their practice and residency, performing CPR and caring for terminal patient experience with a patient who requested DNR affect their views on DNR. In our study, it was observed that there was a significant relationship between the increase in professional experience and positive attitude towards DNR. This finding is consistent with the observation in the study of İyilikçi that less experienced physicians also have less tendency to withdraw treatment (partial or complete).^18^

In our study, most of the physicians who know the legal situation in Turkey, support the DNR request as a right and disapprove of the performing of CPR on all patients. The majority of physicians with experience of having a patient who requests a DNR stated that the DNR order is not abided by practice. Although physicians mostly approve of DNR faced with an ethical dilemma due to the lack of legal regulation. However, (although the legal situation did not change) in the study of İyilikçi et al. at 65.9% of the physicians stated that they gave the DNR order, most of which were verbal^18^. Although physicians' attitudes towards DNR have not changed over time, the main reasons for the change in their behaviour may be that the possibility of encountering legal problems and being subjected to violence from patients' relatives has increased in recent years. In our study, 70.6% of the physicians stated that they were afraid of experiencing legal problems and 45.0% of them were afraid of being exposed to violence from the relatives of the patients. In the study of Periyakoil, when the current data was compared with the period when advance directives became legal and started to become widespread in the USA 23 years ago, although positive views of physicians on advance directives did not change, concerns about the legal situation decreased over time.^22^ Only 62.0's% of physicians working in the units with critically ill patients and often come across patients in the end-of-life period know about the current legal situation on the issue of DNR in Turkey. As the professional experience of the physician increases, the level of knowledge that there is no legal regulation on DNR increases. In a study conducted by Kuvaki et al., this rate was 49% in surgeons.^20^

How to raise the issue of DNR, to make a decision, and who will be involved in the decision-making process is a controversial issue. While the moral situation emphasizes patient autonomy, it becomes difficult to reach a general decision due to the socio-cultural background and the specific conditions and unpredictable consequences of the cases in the practice. Therefore, it seems more appropriate for physicians to prefer a flexible and moderate approach throughout the process rather than a more general approach^6^. The study of Saltbaek et al. pointed out that physicians and patients had disagreements in one out of every three discussions on the DNR decision^31^. In a study in Austria, physicians did not perform CPR 25% of patients who did not have a previous DNR order, considering their age, malignancy, and immediately available patient information.^32^ While DNR decisions are made in advance in cases with poor prognosis such as oncolgy, acute medical situations such as during the Covid-19 pandemic may pose a serious ethical dilemma to the patient and family, physicians and nurses, and the institution.^33^

In the studies, most participants stated that a coalition consisting of the hospital administration, ethics committee, patient and their relatives, and the patient's physician should make the DNR decision but couldn't agree on who would make the final decision.^18^,^31^ Similarly, in our study, while 60.0% of physicians stated that physicians should discuss with patients on a DNR order, 45.7% of them said that patients could make the right decision, and 57.3% of them also stated that the patient should make the final decision. Additionally, 53.5% of the physicians believe that an authorized committee must approve the DNR order of the patient. Some physicians tend to favour the “majority rules” approach. This “None of us is as good as all of us” approach spreads the responsibility as far as possible while also reassuring the physicians on making this critical decision.^34^ However, 24.7% of the physicians in our study were concerned that patients with a DNR decision would not receive adequate care and treatment. In the study of Ur Rahman, 46.5% of the physicians who have the same concern stated that they should provide adequate care and comfort to the DNR patient.^13^ By establishing appropriate care and treatment guidelines, all processes should be managed well with an honest, fair, and transparent approach to eliminate these concerns or prejudices.

Although the participation of physicians is at the forefront in the DNR decision processes, it is stated in the literature that patients and their relatives may sometimes prefer to meet with a nurse or a trained counselor. 35 While in our study, 79% of the physicians stated that patients and their relatives should seek counseling on DNR, 47% of the physicians believed that the opinion of the patient's relative or legal representative is crucial in the DNR decision, only 14.1% emphasized that the thought of the nurses is also considerable. In the study of Granja et al., the rate of physicians who stated that the opinions of nurses are important is 85.6%, however, the majority of the physicians (91.4%) believed that the primary responsibility for the decision of DNR should be the physicians.^36^

We have evaluated physicians' views on DNR within the framework of particular individual characteristics, socio-cultural backgrounds, and professional experiences. The knowledge, skills, and experience that physicians acquire through professional practices are more determinant in their views on DNR than their individual and socio-cultural background, or gains through profession can transform personal and socio-cultural characteristics. Due to the lack of a comprehensive legal regulation on DNR in Turkey, the issue could not discuss effectively, and appropriate guidelines can not be put forward, consequently, physicians have difficulties in this issue and adopt the defensive medicine approach in practice. As a view of defensive medicine, it prefers avoidance in the form of either rejecting the patient's request for the DNR decision or staying out of the process or assurance behavior, which is the attitude of suggesting a DNR decision by distributing responsibility with the broadest coalition. The risk of encountering legal problems and exposure to violence stands out as the main factors in adopting this attitude.

Although most physicians declared that not perform the DNR orders in practice, one of every six of them believed that executed due to the patient's wish. On the other hand, it is also a substantial problem that an application that has not yet been legal according to which criteria, albeit limited, is carried out. The principle of respect for autonomy, which is a fundamental ethical principle, also protects the right of the patient to refuse treatment and request DNR. 37 Physicians have faced a dilemma between their ethical responsibilities and legal obligations regarding DNR in Turkey. According to the Declaration of the World Medical Association on the Relationship between Law and Ethics; Ethical Values and legal principles are usually closely related, but ethical obligations typically exceed legal duties. In some cases, the law mandates unethical conduct. The fact that a physician has complied with the law does not necessarily mean that the physician acted ethically. When law is in conflict with medical ethics, physicians should work to change the law. In circumstances of such conflict, ethical responsibilities supersede legal obligations.^38^

In conclusion, it is essential to make legislation on DNR and end-of-life decisions in Turkey to protect patient autonomy and accept DNR orders as a right. Additionally, the implementation of the Continuing Professional Development Programs on DNR is necessary to increase the ethical sensitivity of physicians as well as to improve their knowledge and skills. There is a need for further research on these issues, mainly qualitative studies.

## Limitations

We conducted the study in a single institution, and there are limitations in the generalizability of the results obtained, as in other studies conducted in a single center. Although the center is one of the two largest hospitals in Turkey's third-largest city, the participants' perspectives may have been influenced by the common view of the single institution. Therefore, future multicenter studies may reduce these limitations.

## Figures and Tables

**Table 2:** Physicians' knowledge and experience with DNR

Knowledge and Experience with DNR	Yes n (%)	No n (%)	I don't recall/ No idea n (%)	p
Is there a legal regulation regarding DNR in our country?	20 (9.9)	127 (62.6)	56 (27.6)	< 0.001
Do you think that the DNR request of the patient / relative is abided by in our country?	29 (14.3)	140 (69.0)	34 (16.7)	< 0.001
Did you take Ethics / Medical Deontology lessons during undergraduate education?	194 (95.6)	9 (4.4)	-	< 0.001
Have you read the literature about DNR?	56 (27.6)	147 (72.4)	-	< 0.001
Have you attended a professional meeting on DNR?	29 (14.3)	174 (85.7)	-	< 0.001
Have you performed CPR on a patient	190 (93.6)	13 (6.4)	-	< 0.001
Have you cared for a terminal patient?	178 (87.7)	25 (12.3)	-	< 0.001
Have you had a patient who requested DNR?	104 (51.2)	99 (48.8)	-	0.726

**Table 3:** Physicians' professional experience and opinions of DNR order in Turkey

		Do you think that the DNR request of the patient / patient's relative is abided by in our country?			p
		Yes	No	No idea	
Have you performed CPR on a patient	Yes	29 (%15,3)	134 (%70,5)	27 (%14,2)	<0.01
	No	0 (%0.0)	6 (%46.2)	7 (%53,8)	
Have you cared for a terminal patient?	Yes	27 (% 15,2)	130 (%73,0)	21 (%11,8)	<0.001
	No	2 (%8,0)	10 (%40.0)	13 (%52,0)	
Have you had a patient who requested DNR?	Yes	17 (%16,3)	81 (%77,9)	6 (%5,8)	<0.001
	No	12 (%12.1)	59 (%59,6)	28 (%28,3)	

**Table 4:** Physicians' Opinions on DNR

	Negative n (%)	Undecided n (%)	Positive n (%)	p
I think CPR is useless in terminal patients	50 (25,1)	38 (19,1)	111 (55,8)	< 0.001
I think the DNR request should be a right for patients	14 (7,0)	38 (19,0)	148 (74,0)	< 0.001
I think the final decision on the DNR should be made by the patient	42 (21,1)	43 (21,6)	114 (57,3)	< 0.001
I think that the palliative care of the patient for whom the DNR decision was made should continue	17 (8,5)	34 (17,1)	148 (74,4)	< 0.001
I think the DNR request of the patient / patient relatives should be abided by.	26 (13,1)	68 (34,2)	105 (52,8)	< 0.001
I think an authorized committee should approve the patient's DNR request.	41 (20,7)	51 (25,8)	106 (53,5)	< 0.001
I think that the patient for whom the DNR decision has been made should be cared for by his/her own physician.	29 (14,6)	61 (30,7)	109 (54,8)	< 0.001
I think CPR should be applied to all patients in case of cardiac / pulmonary arrest.	82 (41,4)	42 (21,2)	74 (37,4)	< 0.01
When the patient / relatives request DNR, i would consider applying slow code to the patient.	83 (43,5)	60 (31,4)	48 (25,1)	< 0.001

**Table 5:** Physicians' Attitudes on DNR

	Negative n (%)	Undecided n (%)	Positive n (%)	p
I would like to recommend DNR to patients / relatives when needed.	43 (21,4)	34 (16,9)	124 (61,7)	< 0.001
I would like the DNR decision to be applied for myself when needed.	24 (11,9)	39 (19,4)	138 (68,7)	< 0.001
I would like the DNR decision to be applied for my relatives when needed.	27 (13,4)	43 (21,4)	131 (65,2)	< 0.001
I would like to consider the age of the patient while making the DNR decision.	35 (17,7)	43 (21,7)	120 (60,6)	< 0.001
I would like to consider the social status or education level of the patient when making the DNR decision.	114 (56,7)	33 (16,4)	54 (26,9)	< 0.001
I would like to consider the nature and prognosis of the disease while making a decision on DNR.	9 (4,5)	29 (14,4)	163 (81,1)	< 0.001
I would like to take into consideration whether the patient is an organ donor while making the DNR decision.	36 (18,0)	39 (19,5)	125 (62,5)	< 0.001
I would like to consider the ward bed occupancy rate while making the DNR decision.	156 (77,6)	12 (6,0)	33 (16,4)	< 0.001
I would like to consider the economic burden of the disease while making a decision for DNR.	132 (65,7)	28 (13,9)	41 (20,4)	< 0.001
I would like the patients or relatives to be counseled by a trained healthcare professional.	14 (7,0)	28 (14,0)	158 (79,0)	< 0.001
I would like there to be legal regulation on DNR.	14 (7,0)	10 (5,0)	177 (88,1)	< 0.001
I would like the DNR as a subject to be in the resident training program.	12 (6,0)	17 (8,5)	172 (85,6)	< 0.001
I do not think DNR decision as correct from my professional point of view.	159 (79,1)	21 (10,4)	21 (10,4)	< 0.001
I do not think DNR decision as correct from my ethical/moral point of views	158 (78,6)	20 (10.0)	23 (11,4)	< 0.001
I do not think DNR decision as correct because of my religious belief.	168 (84,0)	16 (8,0)	16 (8,0)	< 0.001
DNR decision worries me.	93 (46,5)	51 (25,5)	56 (28,0)	< 0.001
I am afraid of verbal/physical violence by patients/' relatives for whom DNR decision is made.	53 (26,5)	57 (28,5)	90 (45,0)	< 0.01
